# Molecular cloning and functional analysis of 4-coumarate: CoA ligases from *Marchantia paleacea* and their roles in lignin and flavanone biosynthesis

**DOI:** 10.1371/journal.pone.0296079

**Published:** 2024-01-08

**Authors:** Shuai Gao, Xin-Yan Liu, Rong Ni, Jie Fu, Hui Tan, Ai-Xia Cheng, Hong-Xiang Lou

**Affiliations:** 1 Key Laboratory of Chemical Biology of Natural Products, Ministry of Education, School of Pharmaceutical Sciences, Shandong University, Jinan, Shandong, China; 2 Shandong Provincial Clinical Research Center for Emergency and Critical Care Medicine, Jinan, Shan-dong, China; University of Sao Paulo, BRAZIL

## Abstract

Phenylpropanoids play important roles in plant physiology and the enzyme 4-coumarate: coenzyme A ligase (4CL) catalyzes the formation of thioesters. Despite extensive characterization in various plants, the functions of 4CLs in the liverwort *Marchantia paleacea* remain unknown. Here, four 4CLs from *M*. *paleacea* were isolated and functionally analyzed. Heterologous expression in *Escherichia coli* indicated the presence of different enzymatic activities in the four enzymes. Mp4CL1 and Mp4CL2 were able to convert caffeic, p-coumaric, cinnamic, ferulic, dihydro-p-coumaric, and 5-hydroxyferulic acids to their corresponding CoA esters, while Mp4CL3 and Mp4CL4 catalyzed none. Mp4CL1 transcription was induced when *M*. *paleacea* thalli were treated with methyl jasmonate (MeJA). The overexpression of Mp4CL1 increased the levels of lignin in transgenic Arabidopsis. In addition, we reconstructed the flavanone biosynthetic pathway in *E*. *coli*. The pathway comprised Mp4CL1, co-expressed with chalcone synthase (CHS) from different plant species, and the efficiency of biosynthesis was optimal when both the 4CL and CHS were obtained from the same species *M*. *paleacea*.

## Introduction

**Fig 1:**
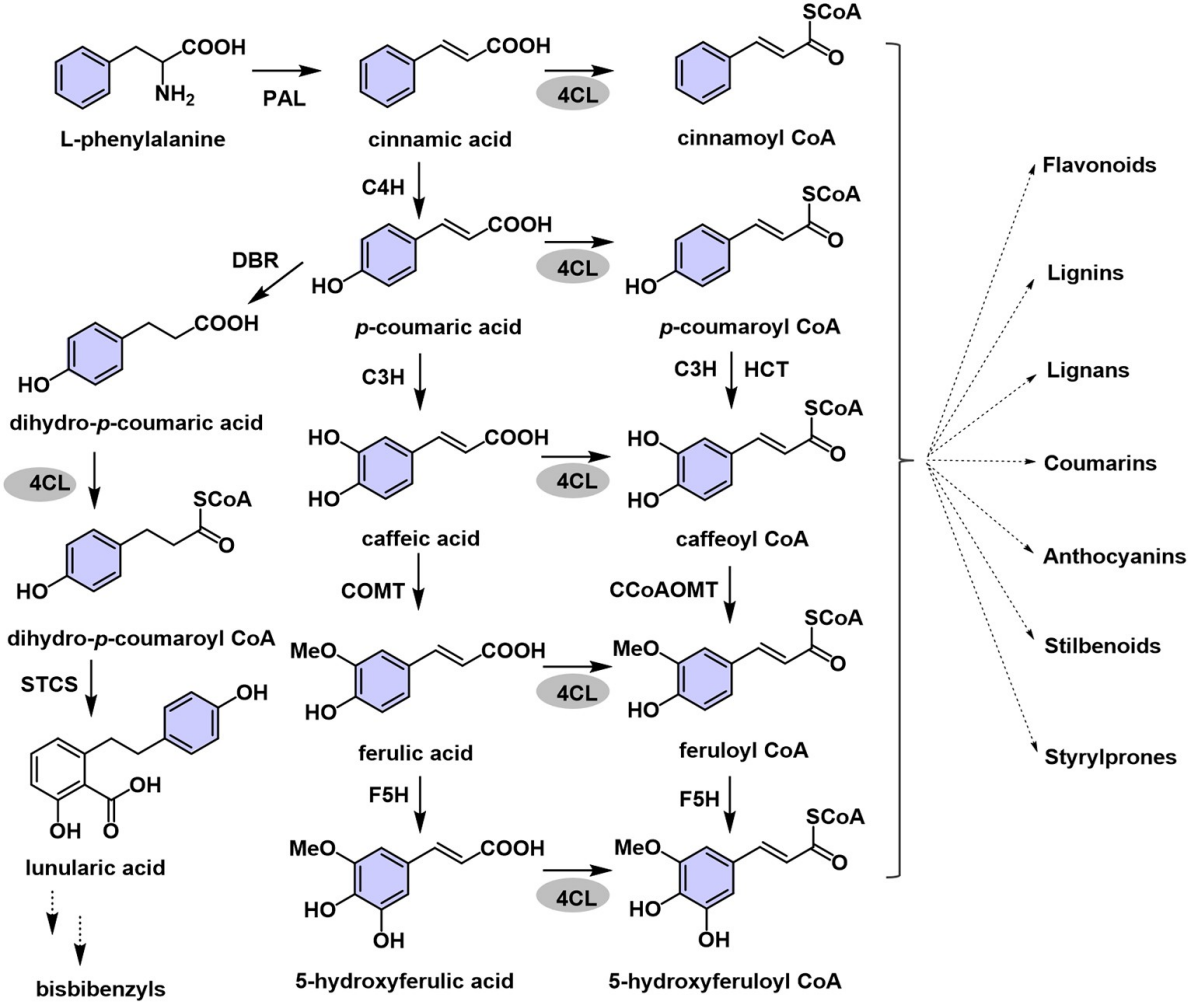
The proposed synthesis pathway of polyphenol compounds in liverworts. PAL, phenylalanine ammonia-lyase; C4H, cinnamate 4-hydroxylase; 4CL, 4-coumarate CoA ligase; C3H, 4-coumarate 3-hydroxylase; COMT, caffeic acid *O*-methyl transferase; F5H, ferulate 5-hydroxylase; HCT, *p*-hydroxy cinnamoyl transferase; CCoAOMT, caffeoyl-CoA *O*-methyltransferase; CHS, chalcone synthase; STCS, stilbenecarboxylate synthase; DBR, double bond reductase.

Phenylpropanoids, an important class of plant secondary metabolites, are widely present in terrestrial plants. Lignins, flavonoids, coumarins, suberins, and bibenzyls are all phenylpropanoid derivatives synthesized via the phenylpropanoid pathway [1]. Phenylpropanoids play vital roles in plants where they protect against various biotic and abiotic stress, such as drought and UV-B radiation [2]. Furthermore, phenylpropanoids possess a variety of pharmacological activities. For example, bibenzyls have decent anti-cancer, anti-fungal, and anti-proliferative activity [3], and flavonoids have anti-cholinesterase and anti-inflammatory [4]. Because of the wide range of biological activities and physiological significance, phenylpropanoids and the phenylpropanoid biosynthetic pathway have attracted increasing attention. During phenylpropanoid synthesis, phenylalanine ammonia-lyase and tyrosine ammonia-lyase non-oxidatively deaminate phenylalanine and tyrosine, respectively, to form cinnamic and *p*-coumaric acids, respectively. 4CL uses p-coumaric, cinnamic, caffeic, ferulic and sinapic acids as substrates for the formation of their corresponding CoA thioesters [5, 6]. The corresponding CoA thioesters act as substrates for the synthesis of essential downstream compounds, such as flavonoids, lignins, and bibenzyls [1] (Fig 1). 4CL is a member of the adenylate-forming enzyme family [7] and 4CL genes have been identified in numerous plant species, including *Arabidopsis thaliana* [8], rice [9], and *Populus trichocarpa* [10]. The main branch point enzyme channels the precursors for different phenylpropanoid-derived end products. Members of the 4CL gene family can be classified into four groups: type I, II, III, and IV. Dicot plants’ 4CL genes are grouped into types I and II, and monocot 4CLs are categorized into types III and IV. Type I and III clusters are mainly involved in monolignol biosynthesis, whereas types II and IV are involved in the biosynthesis of phenylpropanoids other than lignins [9]. The 4CL enzyme has multiple isoforms, each of which has a different substrate affinity. The amino acid residues important for recognizing and accommodating substrates at the active sites of enzymes have been identified and characterized [11]. The 4CL enzymes are critically involved in plant growth, development, and responses to stress, both biotic and abiotic. Upregulation of *4CL* has been demonstrated during plant development and in response to exogenous stimuli, such as wounding, pathogen infection, UV radiation, and exposure to methyl jasmonate [9]. The activation of *4CL* upon elicitor treatment or UV irradiation is coordinated with the activation of phenylalanine ammonia-lyase [12, 13], the first enzyme in the general phenylpropanoid pathway. Apart from participating in lignin biosynthesis, 4CL upregulation in tobacco has been found to increase drought tolerance [14].

4CL products can enter the flavonoid biosynthesis pathway through chalcone synthase (CHS) [15]. Flavonoids such as naringenin, homoeriodictyol, pinocembrin, and eriodictyol are present in numerous edible plants and fruits. They also have a variety of useful biological properties, including antioxidation, anticancer, and anti-inflammatory activities [16]. However, their relatively low concentrations, fluctuations according to season and region, and structural diversity present problems for their supply [16]. Hence, the development of synthetic and molecular engineering techniques for the improvement of flavonoid production is an important consideration.

Bryophytes are small multicellular green plants. They represent an important evolutionary lineage between algae and vascular plants. Bryophytes can be divided into liverworts, mosses, and hornworts. As medicinal plants, liverworts are useful in the treatment of hepatitis and burn infections [17]. These plants are rich in some valuable secondary metabolites, among which phenylpropanoids are a major class [18, 19]. With the continuous discovery of phenylpropanes from liverworts, the exploration of the enzymes involved in their biosynthesis is important. 4CL, as major enzyme in phenylpropanoid synthesis, has only been investigated in three bryophyte species—*Anthoceros agrestis*, *Plagiochasma appendiculatum*, and *Physcomitrium patens* [20–22]. Therefore, identifying more 4CL genes in liverworts is important to explain the distribution and accumulation of different phenylpropanes in liverworts and may assist in the understanding of the phylogeny and origin of plant 4CLs. Here, we first cloned four 4CL genes from the liverwort *Marchantia paleacea* and found that two distinct 4CL enzymes (Mp4CL1 and Mp4CL2) had different substrate specificity. The transient expression of GFP-Mp4CL1 and GFP-Mp4CL2 in *Nicotiana benthamiana* leaves demonstrated that both Mp4CL1 and Mp4CL2 are located in the cytoplasm and nucleus. In particular, Mp4CL1 was found to increase lignin content after heterologous overexpression in Arabidopsis and was induced when an *M*. *paleacea* thallus was subjected to abiotic stress. Furthermore, Mp4CL1 combined with the CHS from *Stenoloma chusanum*, *A*. *thaliana*, or *M*. *paleacea* can produce the naringenin product, which further proves the function of Mp4CL1.

## Materials and methods

### Tissues and reagents

*M*. *paleacea* was cultivated in a greenhouse at 25°C with a 12 h light/12 h dark cycle. Two-month-old thalli were harvested, frozen in liquid nitrogen, and stored at −80°C until use. All reagents and chemicals were from Alfa Aesar (Heysham, UK) or Sigma-Aldrich (St. Louis, USA), unless otherwise specified.

### Sequence analysis

Four putative *4CLs* were identified in the *M*. *paleacea* transcriptome sequencing database (SRP078650). The predicted sequences were aligned with those of 4CLs from other plant species using DNAMAN v.7.0.2 software. A neighbor-joining phylogenetic tree was created with MEGA v5.1 [23], and its robustness was confirmed with 1000 bootstrap replications. The tree was based on 4CL polypeptide sequences (S1 Table).

SWISS-MODEL [24] was used for homology modeling of Mp4CL1 and Mp4CL2 using chain A of *Populus tomentosa* 4CL (PDB accession code: 3NI2) [25] bound to adenosine 5’-(3-(4-hydroxyphenyl) propyl) phosphate (APP), an adenosine 5’-coumaroyl phosphate mimic, as the template. The structures were visualized with PyMol [26].

### DNA isolation, recombinant protein expression, and purification

Total RNA was extracted using the cetyltrimethylammonium bromide (CTAB) method and reverse-transcribed to cDNA [27]. Amplification of the full-length open reading frames (ORFs) of *Mp4CL1-4* was performed using the primer pairs Mp4CL1-Sense/Anti-Sense, Mp4CL2-Sense/Anti-Sense, Mp4CL3-Sense/Anti-Sense, and Mp4CL4-Sense/Anti-Sense (S2 Table). The purified amplicons were inserted into pMD19-T (TaKaRa, Shiga, Japan) cloning vector, then the positive clones were selected and sequenced at Sangon Biotech (Shanghai). The ORFs of *Mp4CLs* were PCR amplified using PrimeSTAR^®^ Max DNA Polymerase (TaKaRa) from pMD19-T-*Mp4CLs* plasmids, using the primer pairs Mp4CL1-primer1/2, Mp4CL2-primer1/2, Mp4CL3-primer1/2, and Mp4CL4-primer1/2 (S2 Table). The restriction enzymes *Kpn* I and *Sal* I were used for the digestion of *Mp4CL1* PCR product, *Bam*H I and *Eco*R I for *Mp4CL2* PCR product, *Kpn* I and *Sac* I for *Mp4CL3* PCR product, and *Bam*H I and *Sal* I for *Mp4CL4* PCR product. The PCR products of *Mp4CLs* and pET32a plasmids were digested with the same restriction enzymes at 37°C for 3 h. After double-digestion with restriction enzymes, the restriction fragments and pET32a plasmids were extracted using a Gel Extraction Kit (Omega Bio-Tek, America). The extracted products were ligated using T4 DNA Ligase at 16°C for 12 h and then transfected into *E*. *coli* strain DH5α. Then the positive clones were selected and sequenced at Sangon Biotech (Shanghai). The resultant plasmids were extracted from the positive clones using a Plasmid Mini Kit (Omega Bio-Tek, America) and transfected into *E*. *coli* strain BL21 (DE3) and selected on LB ampicillin plates. The empty pET32a was also transfected into BL21 (DE3) as a control. Following expression induction with 0.5 mM isopropyl β-D-1-thiogalactopyranoside (IPTG) for 20 h at 16°C, the proteins were purified as previously described [22], separated on 12% SDS-PAGE, and the band visualized with Coomassie Blue R250 [28]. Molecular weights were estimated in comparison with standards (10–170 kDa) (Fermentas, USA).

### Enzyme assays

The enzyme activities of Mp4CLs were assessed by CoA esters formation from various derivatives of cinnamic acid. Assays were conducted for 20 min at 30°C with a reaction volume of 200 μL containing 10 μg of purified protein, 200 μM of substrate (*p*-coumaric, caffeic, ferulic, 5-OH ferulic, cinnamic, or dihydro-*p*-coumaric acids), 5 mM ATP, 300 μM CoA, and 5 mM MgCl_2_, in 200 mM Tris-HCl (pH 7.5). Extracts of *E*. *coli* cells with empty pET32a plasmids were used to replace the protein in the controls. The reaction products were analyzed on the Agilent 1260 HPLC system equipped with a DAD detector (190–950 nm) using a reverse-phase C18 column (XDB-C18, 5 μm; Agilent, USA). The mobile phase A was 1% (v/v) phosphoric acid water and phase B was acetonitrile. The gradient was 0–5 min, 5% B; 5–35 min, 5–25% B; 35–36 min, 25–100% B; 36–45 min, 100% B; and 45–55 min, 5% B. The flow rate was 1 mL/min.

Enzyme activities were detected under different temperatures, pH, and metal cations. In addition, the effect of Mg^2+^ was also assessed at concentrations of 1, 2.5, 5, 10, 20, 100, and 200 mM. Michaelis-Menten kinetics were assessed using GraphPad Prism 6. Varying concentrations of the substrate in 200 mM Tris-HCl buffer were evaluated, with monitoring of UV absorption at one-minute intervals. The wavelengths used for the specific CoA esters were 311 nm (cinnamoyl), 333 nm (*p*-coumaroyl), 346 nm (caffeoyl), 346 nm (feruloyl), and 350 nm (5-hydroxyferuloyl) [29–31]. The kinetics of dihydro-*p*-coumaric acid were determined and the product concentrations were calculated from a standard calibration curve using HPLC.

### Treatment with plant growth regulator and gene expression

Two-month-old *M*. *paleacea* thalli were treated with 1 mM MeJA for 0, 6, 12, 24, 36, or 48 h. Samples were initially placed in liquid nitrogen after collection and RNA extraction and cDNA synthesis was performed as described above. Gene expression was assessed by real-time quantitative PCR using the appropriate primers (S2 Table), using an Eppendorf realplex2 system (Eppendorf, Germany). The 20 μL reaction volume included 1 μg cDNA, 0.5 μM of each primer, and 1×SYBR Green PCR Master mix. *M*. *paleacea* elongation factor 1 alpha (accession number: OR881401) was used as a reference gene which was amplified using the primer pair Mpelongation-F/R.

### Subcellular localization

To investigate the subcellular localization of Mp4CLs, full-length cDNAs of *Mp4CL1* and *Mp4CL2* were amplified with the gene-specific primers listed in S2 Table (attB1-Mp4CL1/attB2-Mp4CL1-GFP, attB1-Mp4CL2/attB2-Mp4CL2-GFP). The PCR products were subcloned into plasmid pDONR207 (Invitrogen, Carlsbad, USA) using the Gateway BP Clonase II enzyme mix (ThermoFisher Scientific) according to the protocol offered and verified by complete gene sequencing. Then, two plasmids were transferred into binary vector pGWB5 (Invitrogen, Carlsbad, USA) using the Gateway LR Clonase II enzyme mix (ThermoFisher Scientific) according to the manufacturer’s protocols. These expression vectors bearing the GFP-Mp4CL1 and GFP-Mp4CL2 constructs were transferred into *Agrobacterium tumefaciens* strain EHA105 using the freeze-thaw method [32]. Transient expression in *N*. *benthamiana* leaves was conducted as described with minor modifications [33, 34]. Specifically, positive transformants were cultured at 28°C overnight, rinsed, and resuspended to an optical density of 1 at 600 nm in transformation buffer [33]. The efficiency of transformation was increased by using a helper *A*. *tumefaciens* strain with a p19 construct [35] with a similar concentration. The two suspensions were mixed and incubated at 25°C for 2 h before syringe infiltration into *N*. *benthamiana* leaves. After two days, the leaves were assessed under confocal microscopy (Zeiss LSM 700 laser confocal microscope, Germany). An argon laser (488 nm for GFP, 550 nm for autofluorescence of chlorophyll) and primary beam-splitting mirrors (458/514 or 488 nm) were used. Processing of images was performed with Zeiss ZEN 2009 software.

### Heterologous expression of *Mp4CL1* in Arabidopsis

The *Mp4CL1* ORF was amplified by PCR using the attB1-Mp4CL1/attB2-Mp4CL1-OE primer pairs (S2 Table). After construction of the pGWB5-Mp4CL1 vector, it was transferred into *A*. *tumefaciens* strain EHA105 [32]. The wild-type (WT) *Arabidopsis thaliana* plants were then transformed by the floral dip method [36]. Positive transformants were obtained after cultivation of the seeds on 1/2 Murashige and Skoog (MS) medium containing 50 mg/L kanamycin. The positive transformants of Mp4CL1 were confirmed by Reverse transcription-polymerase chain reaction (RT-PCR) using Mp4CL1-At-F1/Mp4CL1-At-R1 primer pairs and the *AtActin* gene (NM_001338359.1) was used as a reference with the AtActin-F2/AtActin-R2 primer pairs (S2 Table). Homozygous transgenic lines (i.e., Mp4CL1-OE1) from the T3 generation were selected for further analyses.

### Lignin concentrations and histochemistry

Lignin concentrations were assessed using an acetyl bromide (AcBr) based method [37, 38] and lignin staining was performed using phloroglucinol-HCl color development [39]. Frozen sections with the thickness of 20 μm were cut from the base of the two-month-old *A*. *thaliana* stems using MICROM HM550 temperature frozen section machine under -35°C and incubated in 1 mL of 1% phloroglucinol ethanol HCl solution for 2 min. The sections were then washed, stained in 20% HCl, and evaluated and imaged using an inverted optical microscope (IX71, Olympus) equipped with a 4× objective.

### Synthetic production of flavanones

The CHSs sequences were obtained from pET32a- *MpCHS*, *AtCHS*, and *ScCHS1* by PCR and ligated into pCDFDuet-1 between the *Bam*H I/*Hin*d III, *Bam*H I/*Not* I, and *Hin*d III/*Not* I sites to yield pCDFDuet-1-MpCHS, pCDFDuet-1-AtCHS, and pCDFDuet-1-ScCHS1, respectively. Subsequently, Mp4CL1 was cloned into pCDFDuet-1-MpCHS, pCDFDuet-1-AtCHS, and pCDFDuet-1-ScCHS1 through the *Bgl* II and *Kpn* I sites to generate plasmids p1(pCDFDuet-1-MpCHS-Mp4CL1), p2 (pCDFDuet-1-AtCHS-Mp4CL1), and p3 (pCDFDuet-1-ScCHS1-Mp4CL1). Then, plasmids p1, p2, and p3 were introduced into *E*. *coli* BL21 (DE3) to obtain strains E1, E2, and E3, respectively. All constructs were confirmed using Sanger sequencing and colony PCR. The primers and plasmids used are shown in S2 and S3 Tables, respectively. The engineered strains (E1, E2, and E3) were grown in 10 mL of LB (or TB) medium at 37°C with shaking at 120 rpm. When the OD600 reached 0.4–0.6, the temperature was equilibrated to 16°C, and IPTG was added to the final concentration of 0.5 mM. To produce naringenin, varying concentrations of *p*-coumaric acid (100, 300, 500, and 700 μM) were added, followed by incubation at 16°C for 96 h. One milliliter of the samples was collected at regular intervals (every 12 hours) from the media. After two extractions in equal volume of ethyl acetate, the organic layer was collected and evaporated. The product was then resuspended in 100 μL of 80% (v/v) methanol and analyzed by HPLC. The HPLC parameters were as described by Ni *et al*. [40]. Briefly, samples were analyzed with the Agilent 1260 HPLC system equipped with a reverse-phase XDB-C18 column (4.6 mm × 150 mm, 5 μm, Agilent) under the gradient elution mode at a flow rate of 0.8 mL/min. Mobile phases A and B were water (containing 0.1% glacial acetic acid) and methanol, respectively. A linear gradient of 70% A and 30% B to 20% A and 80% B was supplied over 30 min. When the product was pinocembrin, eriodictyol, or homoeriodictyol, the fermentation conditions and detection method were carried out, as mentioned above.

## Results and discussion

### Isolation of genes, sequencing, and homology modeling

**Fig 2:**
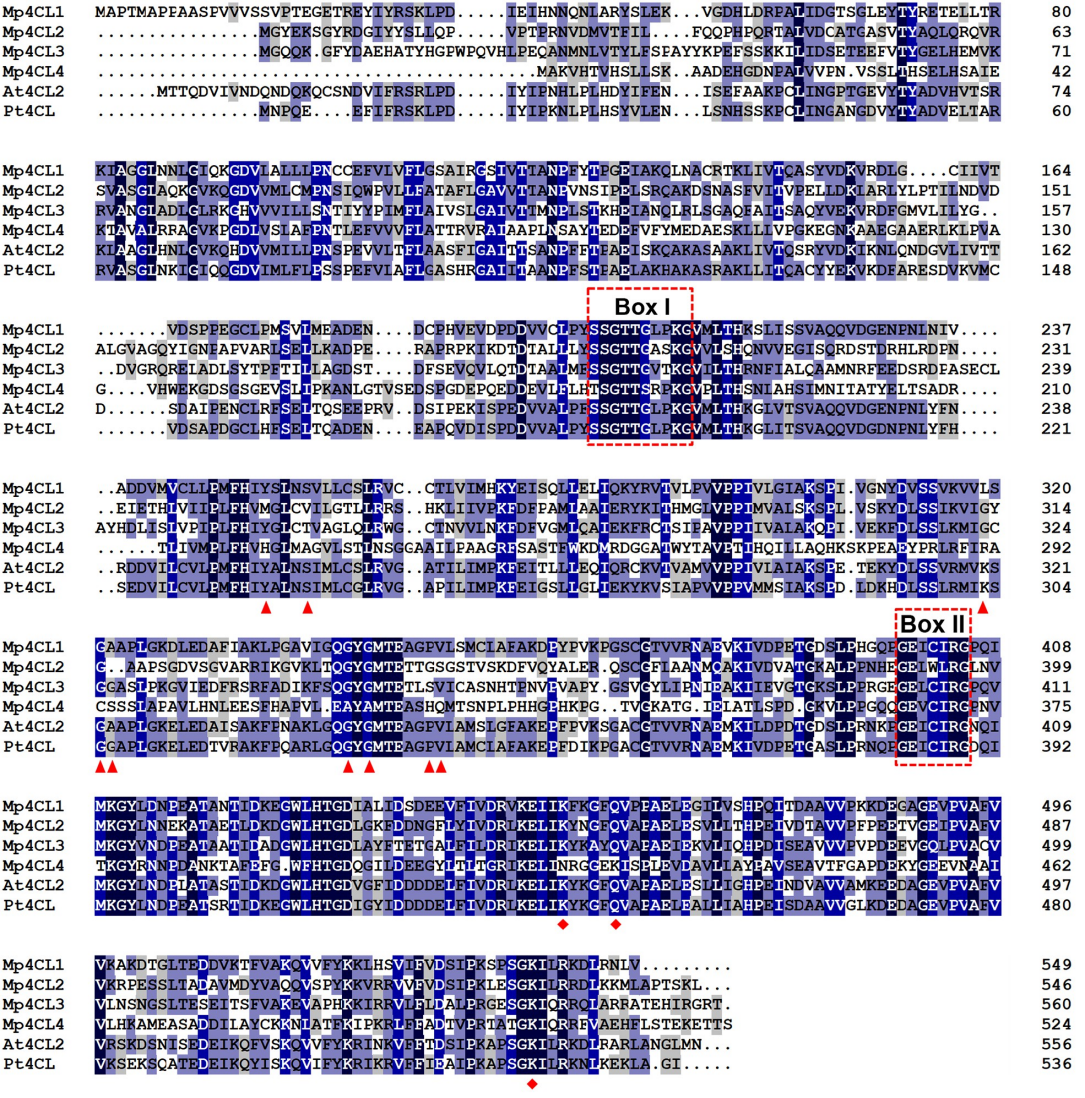
Alignment of the deduced amino acid sequence of *Marchantia paleacea* 4CLs with other plant 4CLs. Residues involved in hydroxycinnamate binding are marked by triangles, while those involved in enzymatic function are marked by diamonds. The putative AMP-binding domain is indicated within Box I, and the conserved “GEICIRG” putative catalytic site is represented in Box II. The abbreviations for species and accession numbers are *Arabidopsis thaliana* 4CL2 (AAD47192.1) and *Populus tomentosa* 4CL (AAL02144.1).

Four putative 4CL genes were identified through mining of the transcriptomic data of *M*. *paleacea* and were designated *Mp4CL1-4*. The open reading frames (ORFs) of the sequences were 1650 (*Mp4CL1*), 1641 (*Mp4CL2*), 1683 (*Mp4CL3*), and 1578 bp (*Mp4CL4*) in length, encoding proteins of 549 (59.21 kDa), 546 (59.60 kDa), 560 (61.57 kDa), and 525 (56.43 kDa) residues, respectively. The protein sequences shared 24.0–59.4% similarity with At4CL2 (accession number: NP_188761) and Pt4CL (accession number: AAL02144). Specifically, Mp4CL1 shared 58.7% identity with At4CL2 and 59.4% identity with Pt4CL, while Mp4CL4 shared 24.0% and 27.3% identity, respectively. All Mp4CLs contained two highly conserved domains, namely, a putative AMP-binding domain (Box I) and the conserved GEICIRG domain (Box II) (Fig 2).

Three-dimensional structures of the Mp4CL1-APP and Mp4CL2-APP complexes were modeled using the 4CL chain A from *P*. *tomentosa* 4CL as the template (S1 Fig). Both structures resembled that of the *P*. *tomentosa* protein in having two globular domains, a larger domain in the N-terminal region and a smaller domain in the C-terminal region [25]. In Mp4CL1, the lengths of the N-terminal and C-terminal domains were 450 (residues 1–450) and 100 residues (residues 450–549), respectively, and 441 (residues 1–441) and 105 residues (residues 442–546) in Mp4CL2, respectively. The overall sequence similarities of Mp4CL1 and Mp4CL2 with the template were 62.8% and 40.4%. Furthermore, the value of Global Model Quality Estimate (GMQE) of the modeled Mp4CL1 was 0.83, and the value of Mp4CL2 was 0.73. The APP-binding site was visible as a cavity in the N-terminal domain (S1 Fig). Residues A322 and G321 in Mp4CL1 and A316 and G315 in Mp4CL2 formed hydrogen bonds with the coumaroyl-AMP conjugate, while K454 and Q459 in Mp4CL1 and K445 and Q450 in Mp4CL2 bound the phosphate group using hydrogen bonds (S1B and S1D Fig).

### Phylogenetic analysis of 4CL homologs

**Fig 3:**
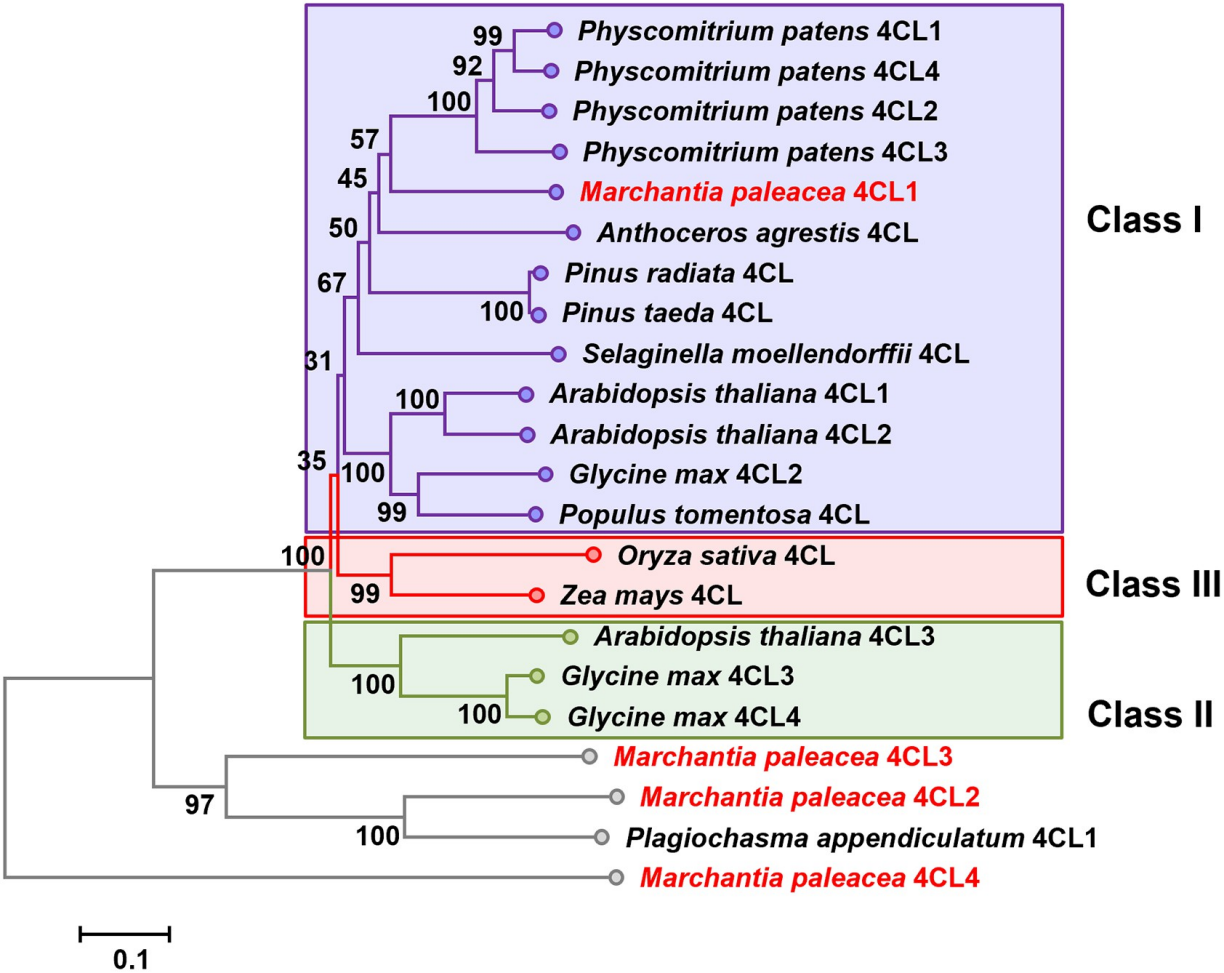
Phylogeny of Mp4CLS and other plant 4CLs. Accession numbers of the sequences are listed in S1 Table.

The neighbor-joining phylogenetic tree was constructed from the protein sequences of *M*. *paleacea*, alfalfa (*Medicago truncatula*), *A*. *thaliana*, soybean (*Glycine max*), *Physcomitrella patens*, and other plant species (GenBank accession numbers listed in S1 Table). After bootstrapping, it could be seen that the 4CL proteins were divided into four subgroups (Fig 3): one clade consisting of Mp4CL2, Mp4CL3, and Pa4CL1 from liverworts; and three clades (classes I, II, and III) of 4CLs that are present in angiosperms, gymnosperms, pteridophytes, and bryophytes (including Mp4CL1) (Fig 3). Class I 4CLs were associated with growth and development, mainly through phenol formation via the lignin pathway, while Class II proteins were linked to abiotic stress responses [41]. Class III 4CLs were predominantly associated with monocotyledonous plants while Class II were found mostly in dicotyledonous species. The tree was rooted with Mp4CL4, indicating that Mp4CL4 might not the real 4CL.

### Enzyme activities HPLC analysis

**Fig 4:**
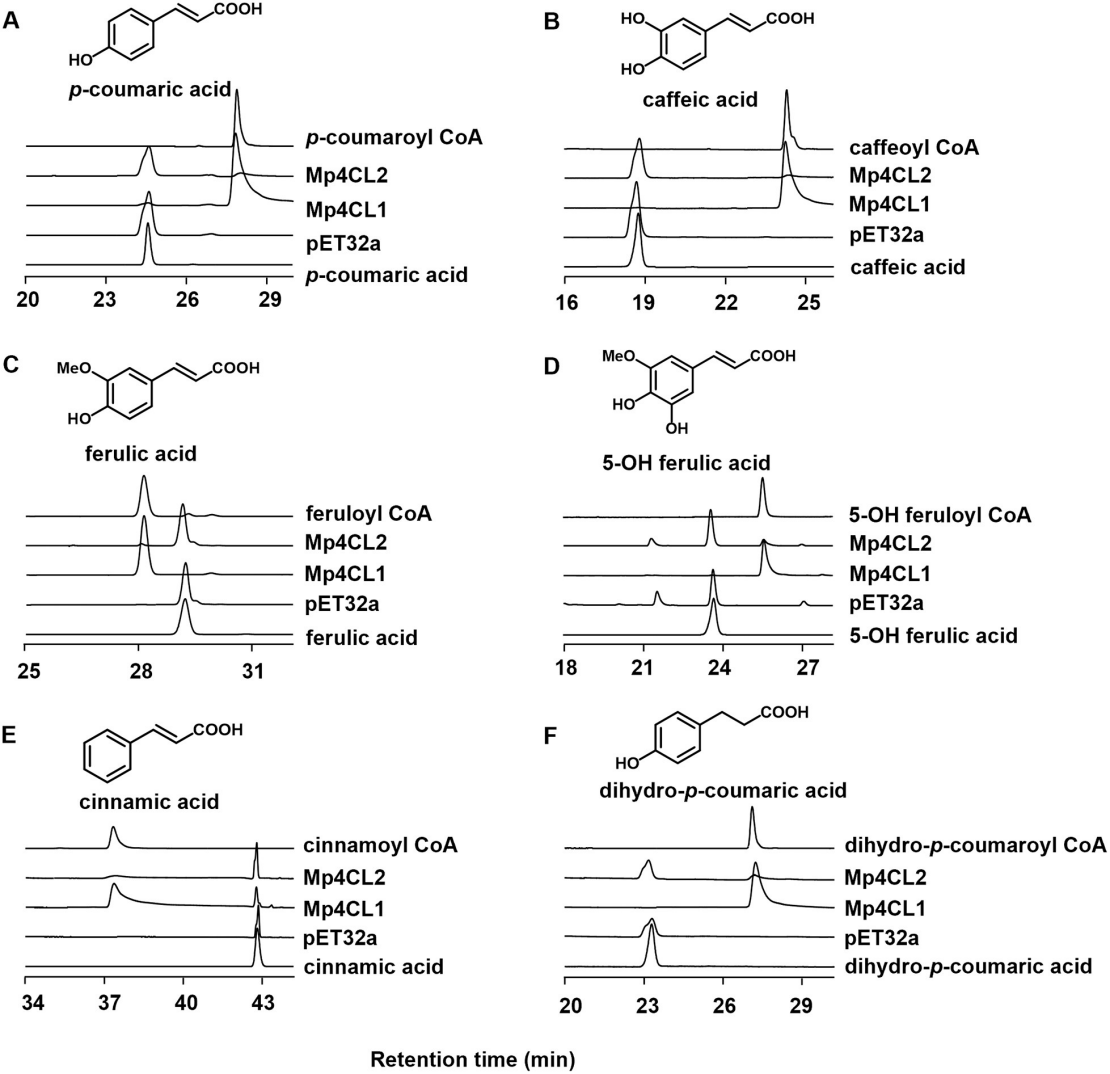
Functional characterization of Mp4CLs. HPLC chromatograms of the reactions of Mp4CLs with *p*-coumaric acid (A), caffeic acid (B), ferulic acid (C), 5-OH ferulic acid (D), cinnamic acid (E), and dihydro-*p*-coumaric acid (F) as substrates.

The *in vitro* enzymatic activities of the expressed Mp4CLs were examined. As shown by SDS-PAGE analysis of the purified recombinant proteins, their molecular weights ranged between 70 and 100 kDa (S2 Fig). The proteins were expressed as fusion proteins with the N-terminal tags, which has a molecular weight of 20.4 kDa. Their activities were assessed using various substrates, including *p*-coumaric, cinnamic, caffeic, ferulic, 5-hydroxy ferulic, and dihydro-*p*-coumaric acids. As shown by HPLC, Mp4CL1 and Mp4CL2 converted all these substrates to their corresponding CoA esters (Fig 4). In contrast, neither Mp4CL3 nor Mp4CL4 displayed any activity with the provided substrates (Table 1). The mechanisms underlying these affinities were explored using modeled structures of Mp4CL1 and Mp4CL2 in complex with APP (S1 Fig). This showed that Y252 and S256 in Mp4CL1, which are necessary for hydroxycinnamate binding and tend to be strongly conserved in 4CL enzymes [25], were replaced by M246 and V250 in Mp4CL2 (S1 Fig). The residues were replaced by H221 and A225 in Mp4CL4, and Y256 and T260 in Mp4CL3, which might explain why Mp4CL3 and Mp4CL4 displayed no activity with the provided substrates. The enzymatic activity of Mp4CL1 for all substrates was higher than that of Mp4CL2 (Table 2). This was likely due to the formation of van der Waals interactions between the respective 4-hydroxyphenyl groups on Y252 and APP in Mp4CL1 as well as the formation of hydrogen bonds between S256 and the hydroxyphenyl group of APP, enhancing the effectiveness of substrate binding.

**Table 1:** The substrate specificity of Mp4CLs.

Substrate	Specific activity (nmol mg^-1^ min^-1^)			
	Mp4CL1	Mp4CL2	Mp4CL3	Mp4CL4
*p*-coumaric acid	124.52±8.76	35.04±0.82	ND	ND
caffeic acid	93.39±10.19	14.29±0.58	ND	ND
ferulic acid	77.44±6.93	12.35±1.15	ND	ND
5-hydroxy ferulic acid	86.69±3.28	4.81±0.19	ND	ND
cinnamic acid	73.32±1.76	26.91±1.22	ND	ND
dihydro-*p*-coumaric acid	176.07±2.12	10.12±0.10	ND	ND

ND, No detectable activity.

**Table 2:** Kinetic parameters of recombinant Mp4CL1.

Substrate	*K*_m_ (μM)	*V*_*max*_ (nmol mg^-1^ min^-1^)	*k*_cat_ (min^-1^)	*k*_*enz*_ (M^-1^min^-1^)
*p*-coumaric acid	93.99±30.94	146.10±17.04	8.65±1.01	9.20×10^4^
caffeic acid	113.30±24.27	165.20±11.00	9.78±0.65	8.63×10^4^
cinnamic acid	115.10±16.56	124.70±5.17	7.39±0.31	6.42×10^4^
ferulic acid	414.10±95.69	364.00±38.71	21.55±2.29	5.20×10^4^
dihydro-*p*-coumaric acid	289.20±36.50	430.1±26.00	25.47±1.54	8.81×10^4^

Furthermore, it was observed that Mp4CL1 activity was sensitive to both pH and temperature. Mp4CL1 was most active at pH 7.0 and 30°C (S3A and S3B Fig). In addition, it is widely known that metal cations also affect enzymatic activity. In our experiment, Mg^2+^ at 10 Mm resulted in the optimum performance (S3C and S3D Fig). We next chose *p*-coumaric, caffeic, cinnamic, ferulic, and dihydro-*p*-coumaric acids for comparison of the kinetic parameters of the enzyme with different substrates. The *V*_*max*_, *K*_*m*_, *k*_*cat*_, and *k*_*enz*_ (*k*_*cat*_*/K*_*m*_) values of Mp4CL1 for the different substrates are shown in Table 2. Mp4CL1 showed the strongest affinity for *p*-coumaric acid (*K*_*m*_ = 93.99 μM), followed by caffeic (*K*_*m*_ = 113.30 μM), cinnamic (*K*_*m*_ = 115.10 μM), dihydro-*p*-coumaric (*K*_*m*_ = 289.20 μM), and ferulic (*K*_*m*_ = 414.10 μM) acids. Additionally, the catalytic efficiency of Mp4CL1 toward *p*-coumaric acid (*k*_*enz*_ = 9.20×10^4^ M^-1^min^-1^) was higher than others. These findings indicated that *p*-coumaric acid was the preferred substrate for Mp4CL1 (Table 2).

### Elicitor-induced Mp4CL1 transcription

**Fig 5:**
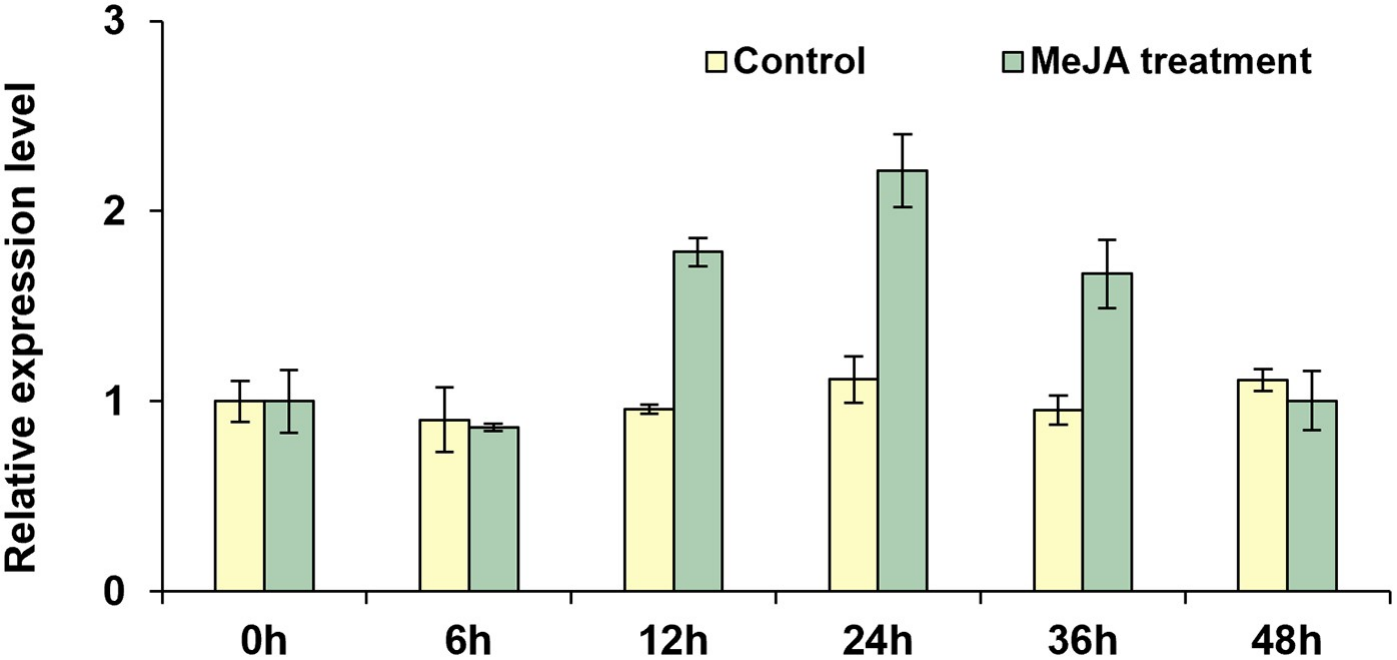
Mp4CL1 transcript abundance in thalli exposed to MeJA. The reference gene was the *Marchantia paleacea* Elongation Factor. The data represent the mean of three biological replicates. Error bars indicate the standard deviation.

It is documented that 4CL enzymes are associated with responses to exogenous signals, such as methyl jasmonate (MeJA), Abscisic acid (ABA) and salicylic acid (SA) [42]. In this research, we measured the expression pattern of *Mp4CL1* under exogenous MeJA treatment using real-time quantitative PCR. As shown in Fig 5, upon MeJA induction, the transcript abundance of *Mp4CL1* in the thallus peaked after 24 h before declining to the levels of the control after 48 h (Fig 5). Similarly, exposure to MeJA upregulated the transcript levels of 4CLs in *Capparis spinosa*, meanwhile, MeJA treatment significantly increased the rutin content of *C*. *spinose* [43]. In the other case, ABA treatment strongly induced the expression of *Cf4CL* of *Coleus forskohlii*, and increased the lignin content [43]. Thus, the present findings indicate the significance of 4CLs, not only in terms of secondary metabolite synthesis, but also in responses to abiotic stressors [44].

### Subcellular localization

**Fig 6:**
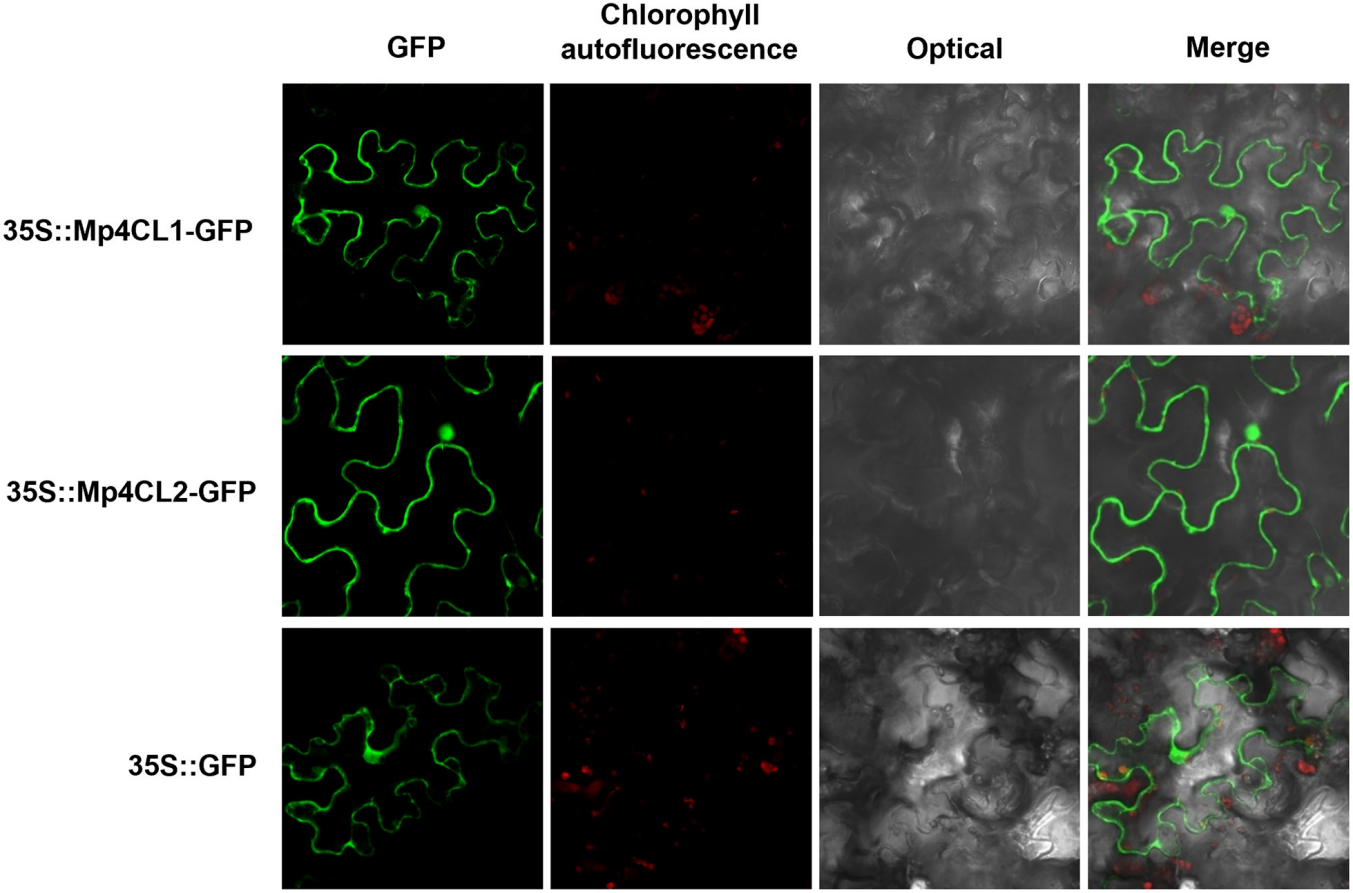
Subcellular localization of *Mp4CLs*. The expression cassettes Mp4CL1- or Mp4CL2-GFP chimeric gene, driven by 35S promoter, transiently transformed tobacco leaf discs. The GFP signal appears green, and the chlorophyll signal is red.

As predicted by SignalP (https://services.healthtech.dtu.dk/services/SignalP-6.0/), the signal peptides of *Mp4CL1* and *Mp4CL2* are located at the N-terminal of proteins, which was the same with some genes the literatures reported [45, 46], the subcellular localization of *Mp4CL1* and *Mp4CL2* was determined *in vivo*. To evaluate the subcellular localization of the *Mp4CL1 and Mp4CL2* proteins, two C-terminal GFP-fusion plasmids (GFP-Mp4CL1 and GFP-Mp4CL2) were constructed and transiently expressed in tobacco leaves. GFP signals were detected in both the nucleus and cytoplasm for the GFP control, GFP-Mp4CL1, and GFP-Mp4CL2 (Fig 6). This result indicates that both Mp4CL1 and Mp4CL2 are localized to the cytoplasm and nucleus, as reported for Cs4CLs [47].

### Mp4CL1 overexpression in arabidopsis

**Fig 7:**
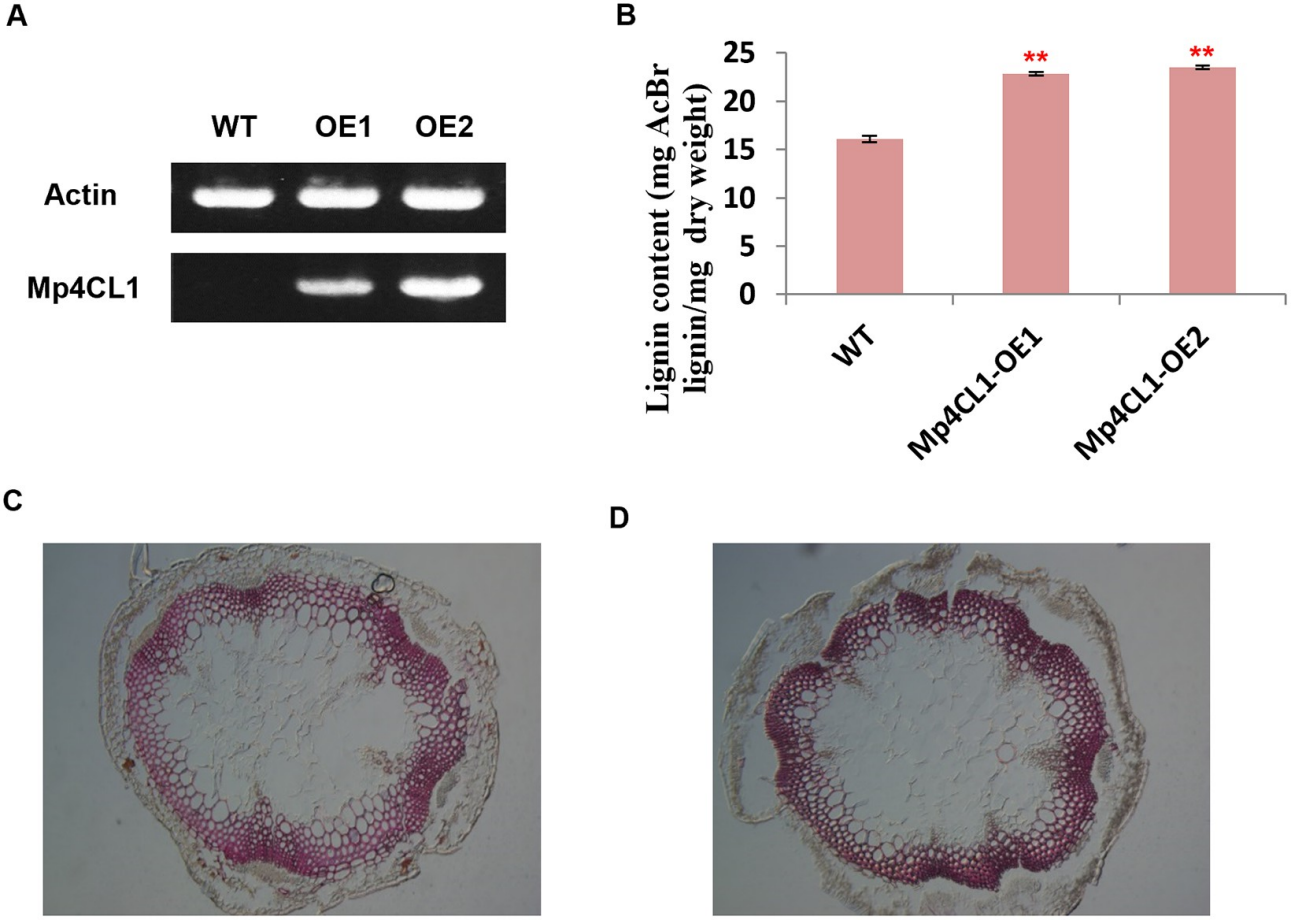
Heterologous expression of *Mp4CL1* genes in *A*. *thaliana* and its effect on lignin content. A. RT-PCR profiling shows that Mp4CL1 was transcribed in the selected transgenic lines; AtActin was used as the reference sequence. B. Lignin content of Mp4CL1 transgenic *A*. *thaliana*. The results were shown as the means of three biological replicate reactions with standard deviations, significant differences were determined by Student’s t-test, *p < 0.05 and **p < 0.01. Phloroglucinol-HCl stained stem sections with the thickness of 20 μm sampled from (C) wild type *A*. *thaliana* plant and (D) transgenic plant expressing 35S::Mp4CL1. Lignin was stained violet-red.

To further functionally characterize Mp4CLs, we overexpressed *Mp4CL1* in Arabidopsis. After insertion of the full-length *Mp4CL1* coding sequence into the pGWB5 vector to generate an overexpression construct and transformation with *A*. *tumefaciens*, lines stably overexpressing Mp4CL1 were generated in the Columbia-0 background. The presence of *Mp4CL1* transcripts in the T3 plants but not in the WT was confirmed by RT-PCR (Fig 7A). The lignin contents of the two lines (22.86 and 23.49 mg AcBr lignin/mg dry weight) were measured and compared with the wild-type control (16.12 mg AcBr lignin/mg dry weight) using the AcBr based method [37, 38]. The result showed that the presence of the two transgenes increased the deposition of lignin by 41.8% and 45.7%, respectively (Fig 7B). As 4CLs are closely involved in lignin biosynthesis [48, 49], lignin deposition was examined in the stems of *Mp4CL1*-OE and WT plants using histochemistry. This showed elevated lignin contents in the stem vascular tissues (Fig 7C and 7D) in the transgenic plants but not the WT. This demonstrates that *Mp4CL1* overexpression enhanced lignin deposition and may thus increase lodging resistance, an important agronomic characteristic.

### Flavanone production in *E*. *coli* from supplementation with different hydroxycinnamic acids using 4CL and CHS enzymes

**Fig 8:**
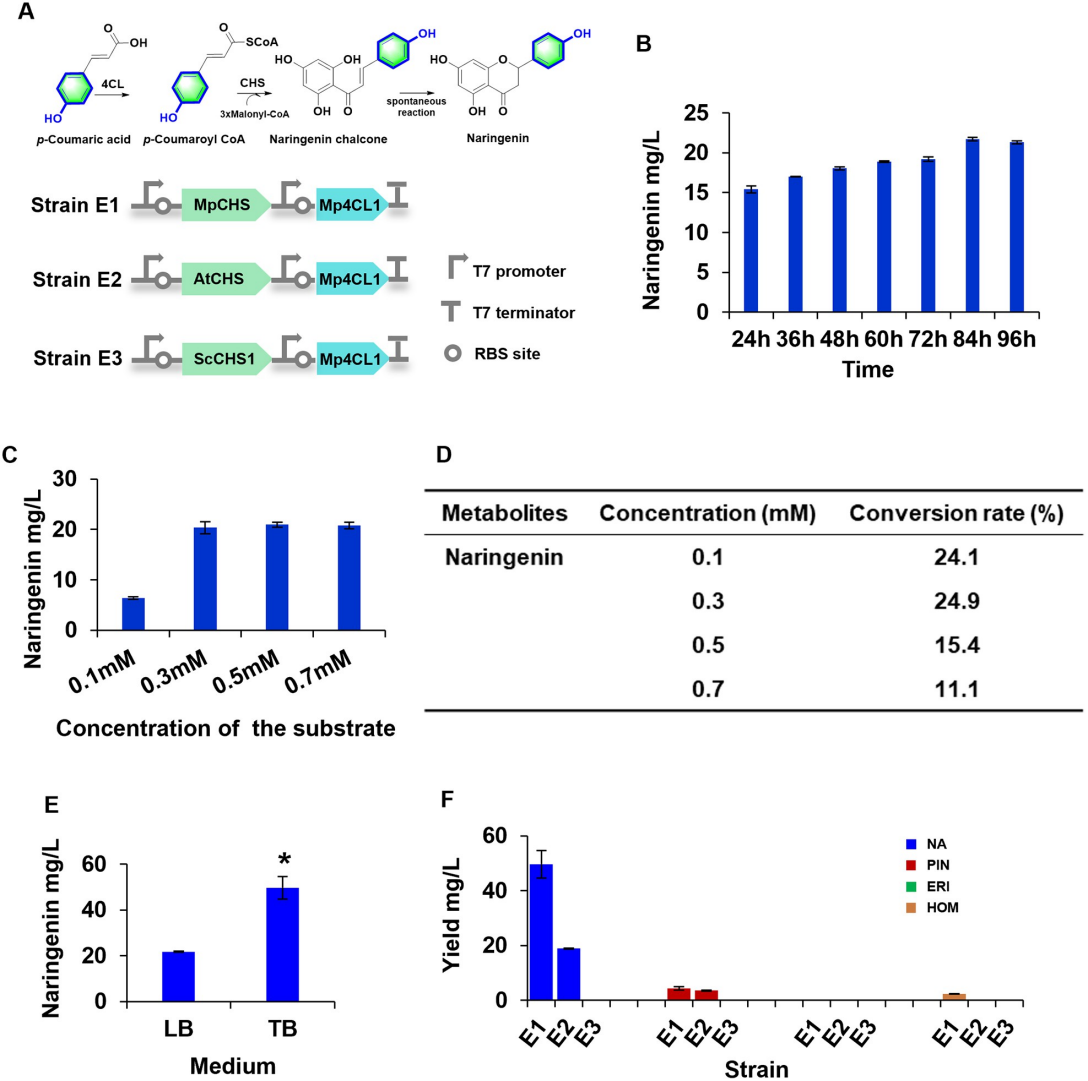
Synthetic production of various flavanones by recombinant *Escherichia coli* strains. A. Engineered pathway for naringenin biosynthesis from *p*-coumaric acid using 4-Coumarate: coenzyme A ligase (4CL) and chalcone synthases (CHS) in *E*. *coli*. B. The effect of fermentation time on the production of naringenin using strain E1. C. Effect of varying concentrations of *p*-coumaric acid on the production of naringenin using strain E1. D. Maximizing the conversion rate of naringenin by optimizing the substrate concentration and time course. E. Effect of optimized (LB and TB) media on naringenin production using strain E1. F. Biosynthesis of naringenin (NAR), pinocembrin (PIN), eriodictyol (ERI), and homoeriodictyol (HOM) in different *E*. *coli* strains. Three biological replicates were performed for each analysis; the error bars indicate the SD.

During flavonoid biosynthesis, both 4CL and chalcone synthase (CHS) are necessary for producing flavanone from various hydroxycinnamic acids. Previous studies have suggested that naringenin biosynthesis from *p*-coumaroyl CoA, which is catalyzed by CHS, is the limiting step for flavonoid production [50]. To date, many CHSs have been characterized in plant species spanning Bryophyta [51], Pteridophyta [40, 52], Gymnospermae, and Angiospermae [53, 54]. In this study, we selected the chalcone synthases MpCHS (AUG98250.1) [55], *Stenoloma chusanaum* chalcone synthase (ScCHS1, ON462260) [40], and AtCHS (NP_196897.1) [56] for investigation by constructing three engineered strains, E1 (harboring *MpCHS* and *Mp4CL1*), E2 (harboring *AtCHS* and *Mp4CL1*), and E3 (harboring *ScCHS1* and *Mp4CL1*) (Fig 8A). Thus, Mp4CL1, which displayed high enzyme activity *in vitro* (Fig 4), was used to convert *p*-coumaric acid to *p*-coumaroyl CoA. As shown in Fig 8, after 84 h of fermentation, strain E1 produced naringenin at a concentration of 21 mg/L (supplemented with 500 μM of *p*-coumaric acid) at a rate of 15.4% in LB media (Fig 8B and 8D). For optimization, strain E1 was supplemented with substrate at different concentrations. Via HPLC analysis, the optimal substrate concentration was determined to be 300 μM (Fig 8B); at this concentration, the maximum conversion rate was as high as 24.9% (Fig 8C and 8D). Under optimized conditions, strains E1, E2, and E3 were suspended in the TB medium with 300 μM of *p*-coumaric acid, cinnamic acid, caffeic acid, and ferulic acid for 84 h to produce naringenin, pinocembrin, eriodictyol, and homoeriodictyol respectively. Strain E1 produced approximately 49 mg/L naringenin (conversion yield, 60.8%), 4 mg/L pinocembrin (conversion yield, 5.6%), and 2 mg/L homoeriodictyol (conversion yield, 2.6%), with no eriodictyol being detected in the extract (Fig 8E and 8F; S4 Table). For E2, 18 mg/L of naringenin (conversion yield, 23.1%), 3.5 mg/L of pinocembrin (conversion yield, 4.6%), and no homoeriodictyol and eriodictyol were detected in the extract (Fig 8F; S4 Table). No production was detected in strain E3. As AtCHS and ScCHS1 are from species other than *M*. *paleacea*, there results suggest that flavanone production is likely to be more efficient when 4CL and CHS from the same species are present. Similarly, co-expression of the upstream OsF2H with OsCGT from *Oryza sativa* in yeast was found to maximize the flavone *C*-glucoside yield [57].

## Conclusions

Here, two 4CL genes *Mp4CL1* and *Mp4CL2* were functionally characterized from the liverwort *M*. *paleacea*. They could catalyze *p*-coumaric, caffeic, ferulic, cinnamic, 5-hydroxy ferulic, and dihydro-*p*-coumaric acids to the corresponding hydroxycinnamoyl CoA thioesters. In addition, we investigated the subcellular localization and expression pattern of *Mp4CL1* and *Mp4CL2* in plants treated with MeJA. Mp4CL1 can be used to produce various flavanones in *E*. *coli*. Our research will contribute to the understanding of the functional diversity of 4CL in liverworts.

## Supporting information

S1 Dataset (XLSX)

S1 Fig Homology modeling and docking of Mp4CLs with APP. A. Three-dimensional structural model of the Mp4CL1-APP complex. The structure was obtained by homology modeling using *P*. *tomentosa* 4CL-3NI2 as a template. The N-domain is colored green, and the C-domain is cyan. APP is indicated by a sphere, with its C, O, and N atoms colored in yellow, pink, and blue, respectively. B. Stereoview of the Mp4CL1-APP interaction. The APP C, O, and N atoms are colored in green, pink, and blue, respectively. Hydrogen bonds between APP and Mp4CL1 are shown as red dashed lines. C. Three-dimensional structural model of the Mp4CL2-APP complex. D. Stereoview of the Mp4CL2-APP interaction.
(TIF)

S2 Fig SDS-PAGE analysis of recombinant proteins. M: Weight marker, Lane 1: Mp4CL1, Lane 2: Mp4CL2, Lane 3: Mp4CL3, and Lane 4: Mp4CL4.
(TIF)

S3 Fig Enzymatic characteristics of purified recombinant Mp4CL1. Effect of various pH (**A**), temperature (**B**), mental cations (**C**), and the Mg^2+^ concentration (**D**) on the enzyme activities of Mp4CL1 using *p*-coumaric acid as the substrate.
(TIF)

S1 Table Accession numbers of amino acid sequences used for phylogenetic reconstruction. (DOC)

S2 Table Primers were used in this research. (DOC)

S3 Table Plasmids and strains were used in this study. (DOC)

S4 Table The conversion rate generated by the four hydroxycinnamic acids to produce naringenin, pinocembrin, eriodictyol, and homoeriodictyol at the 300 μM substrate concentration in the TB medium. (DOC)

S1 Raw images (PDF)
