# Supplementary figures and images for: Molecular cloning and functional analysis of 4-coumarate: CoA ligases from Marchantia paleacea and their roles in lignin and flavanone biosynthesis
Source: PLoS One. 2024 Jan 8;19(1):e0296079. doi: 10.1371/journal.pone.0296079 (PMC10773943; doi:10.1371/journal.pone.0296079)

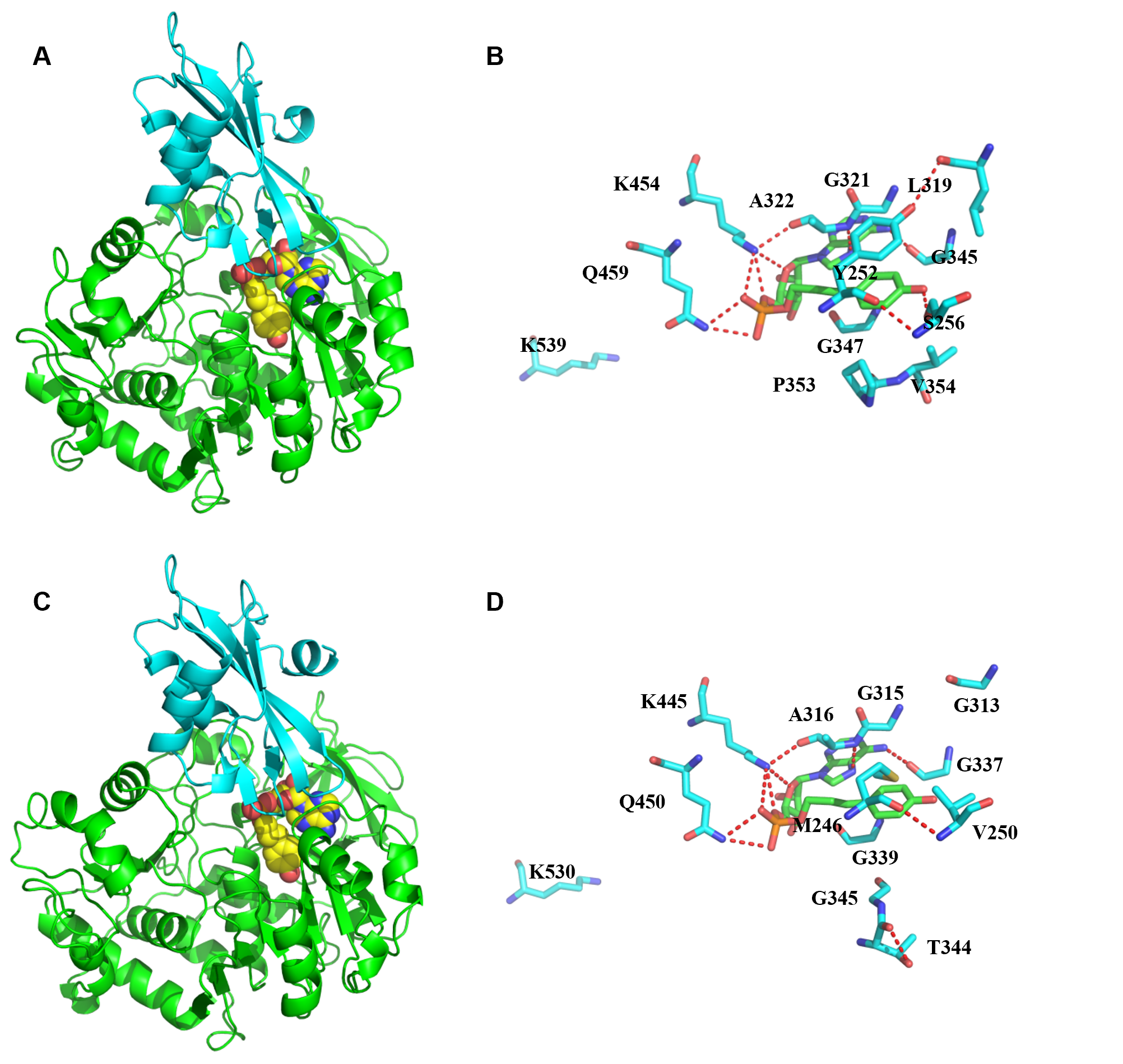

Supplement: S1 Fig — A. Three-dimensional structural model of the Mp4CL1-APP complex. The structure was obtained by homology modeling using P. tomentosa 4CL-3NI2 as a template. The N-domain is colored green, and the C-domain is cyan. APP is indicated by a sphere, with its C, O, and N atoms colored in yellow, pink, and blue, respectively. B. Stereoview of the Mp4CL1-APP interaction. The APP C, O, and N atoms are colored in green, pink, and blue, respectively. Hydrogen bonds between APP and Mp4CL1 are shown as red dashed lines. C. Three-dimensional structural model of the Mp4CL2-APP complex. D. Stereoview of the Mp4CL2-APP interaction. (TIF) [file pone.0296079.s002.tif]

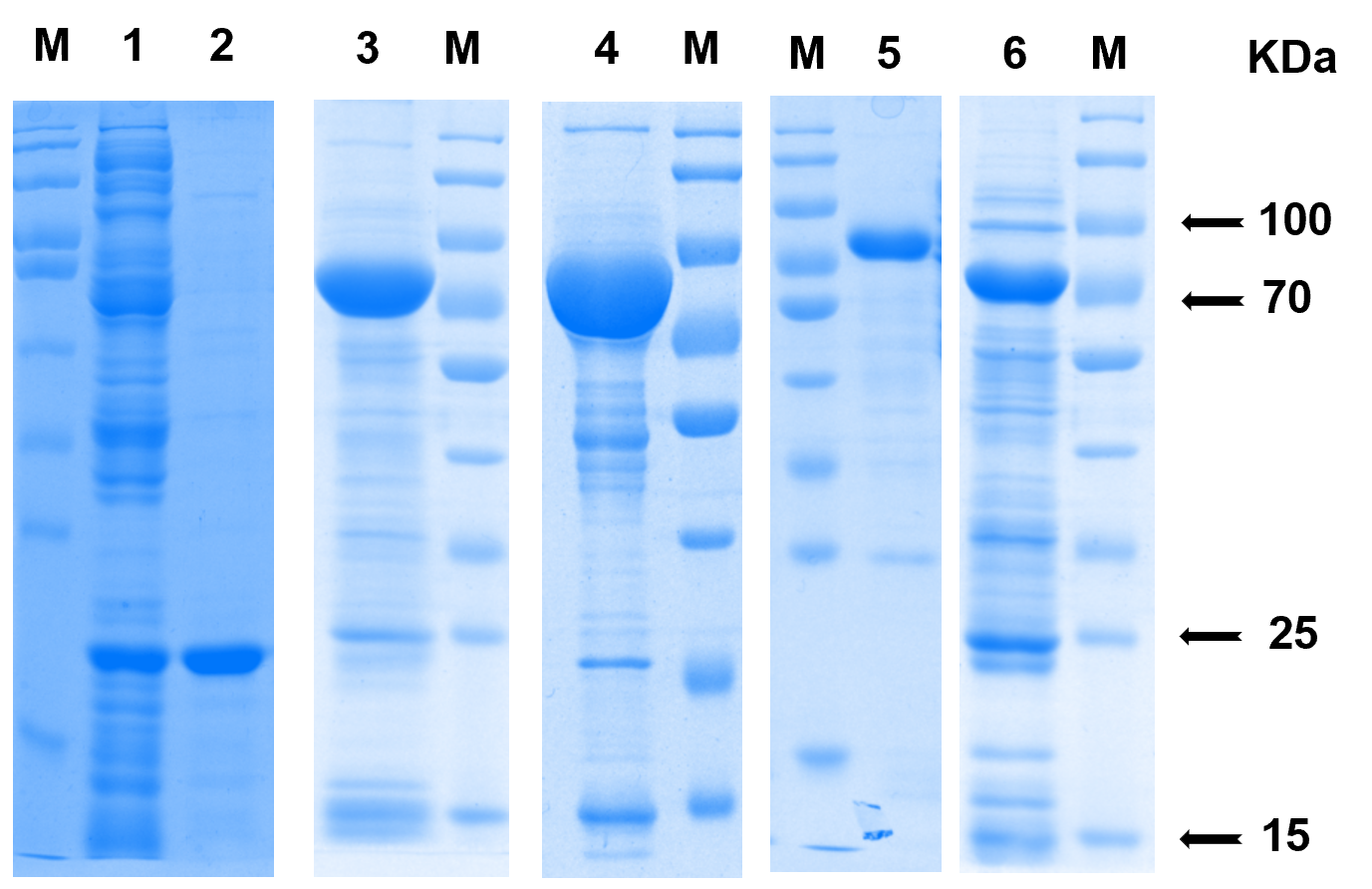

Supplement: S2 Fig — M: Weight marker, Lane 1: Mp4CL1, Lane 2: Mp4CL2, Lane 3: Mp4CL3, and Lane 4: Mp4CL4. (TIF) [file pone.0296079.s003.tif]

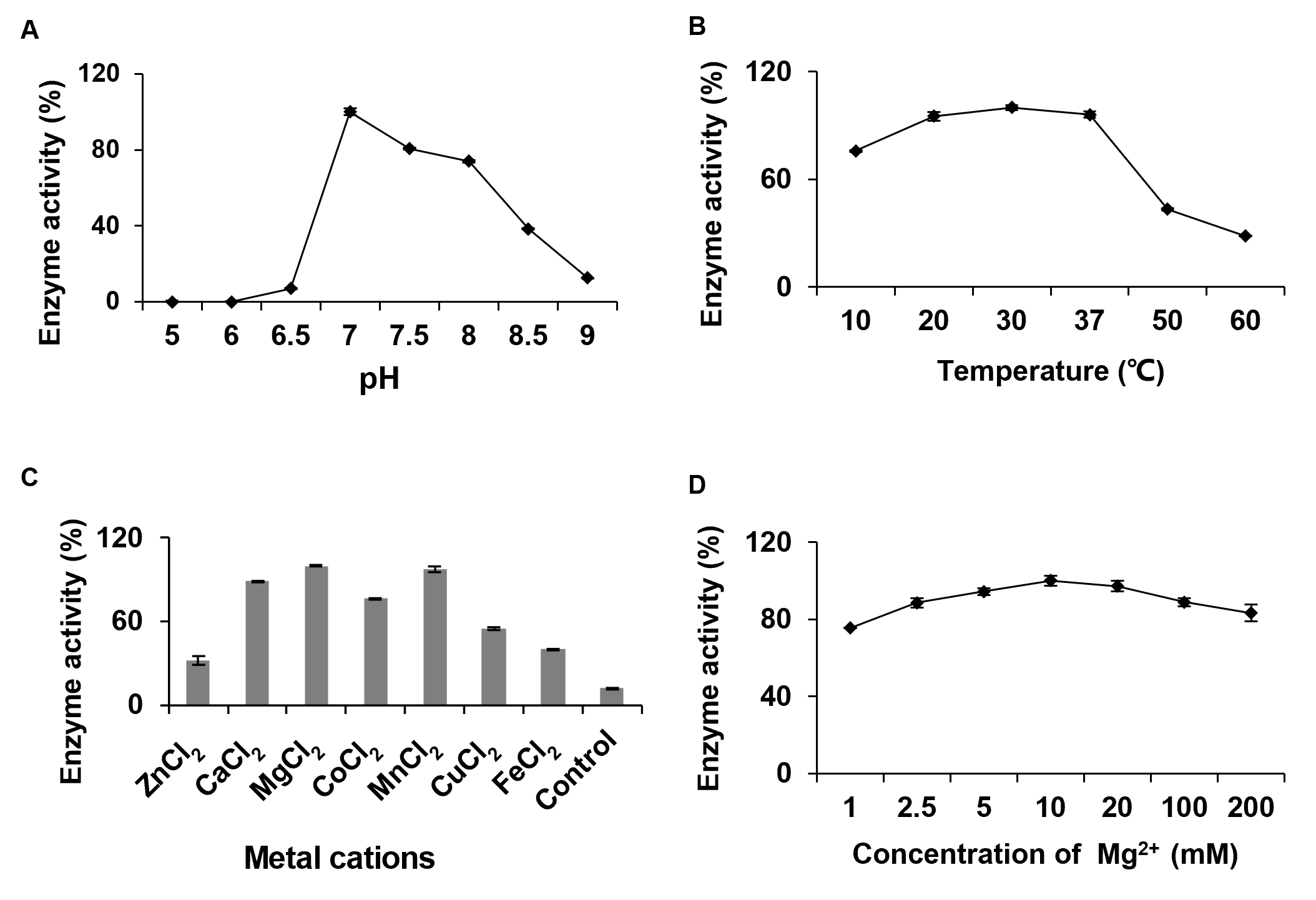

Supplement: S3 Fig — Effect of various pH (A), temperature (B), mental cations (C), and the Mg2+ concentration (D) on the enzyme activities of Mp4CL1 using p-coumaric acid as the substrate. (TIF) [file pone.0296079.s004.tif]

Fig 7A

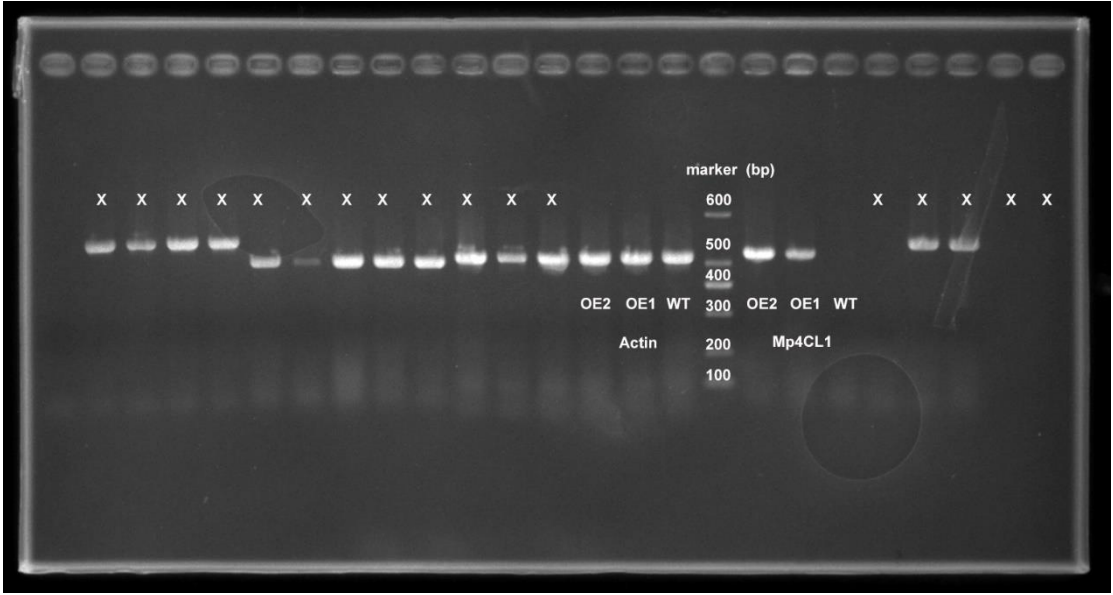

Fig S2

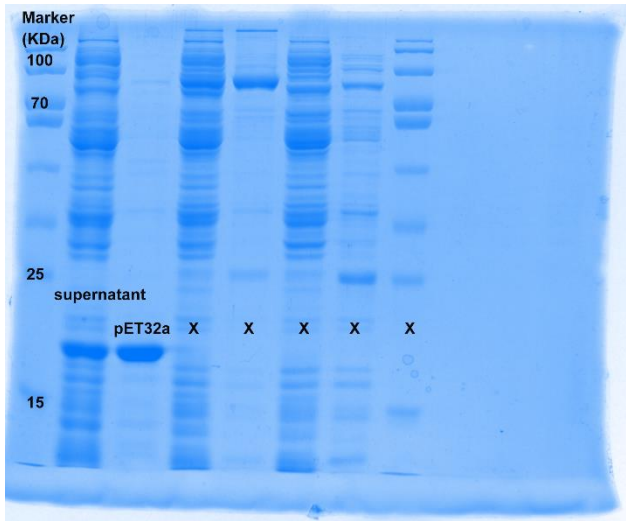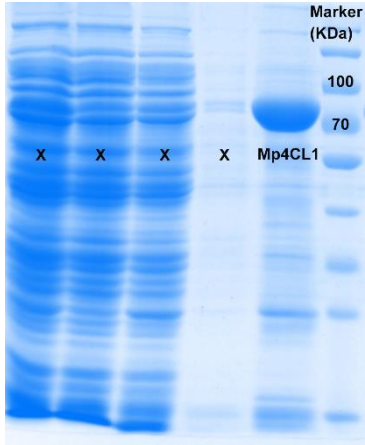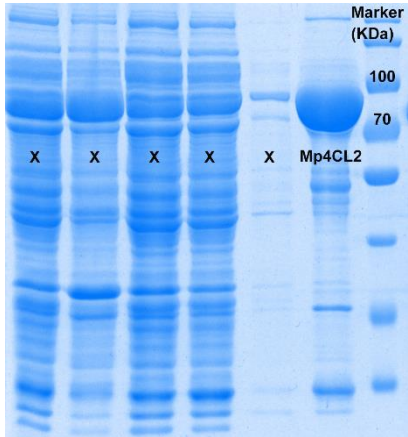

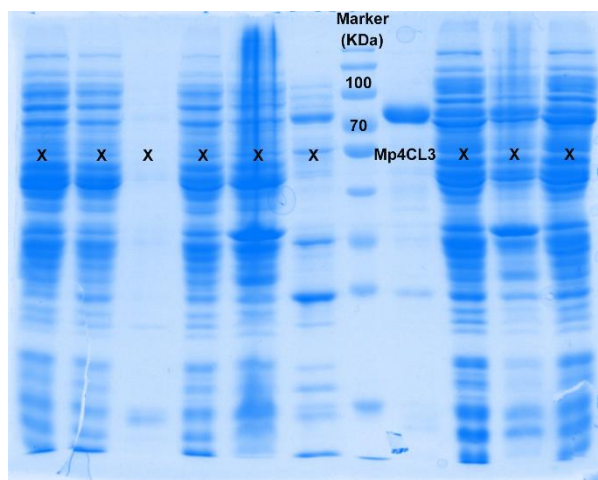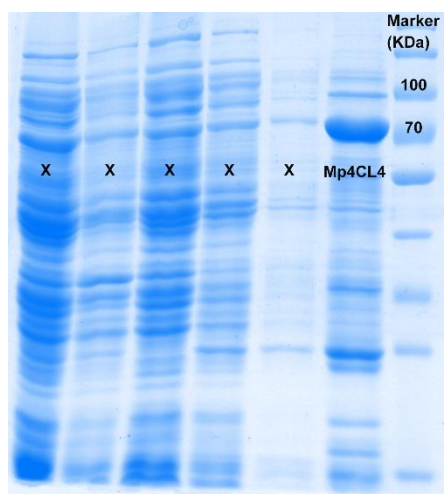

Supplement: S1 Raw images — (PDF) [file pone.0296079.s009.pdf]
