# Supplementary material for: Molecular cloning and functional analysis of 4-coumarate: CoA ligases from Marchantia paleacea and their roles in lignin and flavanone biosynthesis
Source: PLoS One. 2024 Jan 8;19(1):e0296079. doi: 10.1371/journal.pone.0296079 (PMC10773943; doi:10.1371/journal.pone.0296079)
Supplement: S1 Table — (DOC) [file pone.0296079.s005.doc]

**Supporting information**

**S1 Table. Accession numbers of amino acid sequences used for phylogenetic reconstruction.**

| Sequence | Accession No. |
| --- | --- |
| *Physcomitrium patens* 4CL1 | ABV60447 |
| *Physcomitrium patens* 4CL4 | ABV60450 |
| *Physcomitrium patens* 4CL2 | ABV60448 |
| *Physcomitrium patens* 4CL3 | ABV60449 |
| *Marchantia paleacea* 4CL1 | OQ718878 |
| *Anthoceros agrestis* 4CL | QJX14324 |
| *Pinus radiata* 4CL | ACF35279 |
| *Pinus taeda* 4CL | AAB42383 |
| *Selaginella moellendorffii* 4CL | XP_002969881 |
| *Arabidopsis thaliana* 4CL1 | NP_001077697 |
| *Arabidopsis thaliana* 4CL2 | NP_188761 |
| *Populus tomentosa* 4CL | AAL02144 |
| *Glycine max* 4CL2 | NP_001236418 |
| *Oryza sativa* 4CL | CAA36850 |
| *Zea mays* 4CL | AAS67644 |
| *Arabidopsis thaliana* 4CL3 | NP_849844 |
| *Glycine max* 4CL3 | AAC97389 |
| *Glycine max* 4CL4 | NP_001236236 |
| *Marchantia paleacea* 4CL2 | OQ718879 |
| *Marchantia paleacea* 4CL3 | OQ718880 |
| *Marchantia paleacea* 4CL4 | OQ718881 |
| *Plagiochasma appendiculatum* 4CL1 | KJ944317 |

|  |  |
| --- | --- |
|  |  |
|  |  |
|  |  |
|  |  |
|  |  |
|  |  |
|  |  |
|  |  |
|  |  |
|  |  |
|  |  |
|  |  |
|  |  |
|  |  |
|  |  |
|  |  |
|  |  |
|  |  |
|  |  |
|  |  |
|  |  |
|  |  |
|  |  |
|  |  |
|  |  |
|  |  |
|  |  |
|  |  |
|  |  |
|  |  |
|  |  |
|  |  |
|  |  |
|  |  |
|  |  |
|  |  |

|  |  |
| --- | --- |
|  |  |
|  |  |
|  |  |
|  |  |
|  |  |
|  |  |
|  |  |
|  |  |
|  |  |
|  |  |
|  |  |
|  |  |
|  |  |
|  |  |
|  |  |

|  |  |  |  |
| --- | --- | --- | --- |
|  |  |  |  |
|  |  |  |  |
|  |  |  |  |
|  |  |  |  |
|  |  |  |  |
|  |  |  |  |
|  |  |  |  |
|  |  |  |  |
|  |  |  |  |
|  |  |  |  |
|  |  |  |  |
|  |  |  |  |
