# Supplementary material for: Molecular cloning and functional analysis of 4-coumarate: CoA ligases from Marchantia paleacea and their roles in lignin and flavanone biosynthesis
Source: PLoS One. 2024 Jan 8;19(1):e0296079. doi: 10.1371/journal.pone.0296079 (PMC10773943; doi:10.1371/journal.pone.0296079)
Supplement: S2 Table — (DOC) [file pone.0296079.s006.doc]

**Supporting information**

|  |  |
| --- | --- |
|  |  |
|  |  |
|  |  |
|  |  |
|  |  |
|  |  |
|  |  |
|  |  |
|  |  |
|  |  |
|  |  |
|  |  |
|  |  |
|  |  |
|  |  |
|  |  |
|  |  |
|  |  |
|  |  |
|  |  |
|  |  |
|  |  |

**S2 Table. Primers were used in this research.**

| Primer name | Primer sequences (5' to 3') |
| --- | --- |
| Mp4CL1-Sense | GGGAAAGAGAAATGGCGCCTAC |
| Mp4CL1-Anti-Sense | TGGTCTTCGGTGGTGCTGTAGC |
| Mp4CL2-Sense | TCGGTGAAGTTGATAGGCGTT |
| Mp4CL2-Anti-Sense | GCGATATTGTTCAGCGAGGAC |
| Mp4CL3-Sense | GGATAGATCAGGGGTGGATAGTG |
| Mp4CL3-Anti-Sense | TGTGTAGAGCAGATGAAGGGAAA |
| Mp4CL4-Sense | TGATCATTGCTTGTGCTGGTTG |
| Mp4CL4-Anti-Sense  Mp4CL1-primer1  Mp4CL1-primer2  Mp4CL2-primer1 | TCCCCTCTGCTCAGAAGTCTAC  GGGGTACCATGGCGCCTACAATGGCTCC  GCGTCGACCTAAACGAGGTTTCTTAAGT  CGGGATCCATGGGTTACGAGAAGAGTGG |
| Mp4CL2-primer2 | CGGAATTCTCACAATTTCGAAGTAGGAG |
| Mp4CL3-primer1 | GGGGTACCATGGGTCAACAGAAAGGTTT |
| Mp4CL3-primer2 | CCGAGCTCTTAGGTTCTTCCTCGTATGT |
| Mp4CL4-primer1 | CGGGATCCATGGCCAAAGTTCATACCGT |
| Mp4CL4-primer2 | GCGTCGACCTAACCTGAAGTTGTCTCCT |
| Mp4CL1-RT-F2 | CGGGACTGTTGTCAGAAATG |
| Mp4CL1-RT-R2 | GAAGCCATCCCTCCTTATCA |
| Mpelongation-F | GGTGAAGGATCTGAAGCGGGG |
| Mpelongation-R | ACTGCGATGTGCGAGGTGTGG |
| Mp4CL2-RT-F2 | CGTCAGTCCTGTGGGTTCCTA |
| Mp4CL2-RT-R2 | CAGTTGCCTTCTCGTTGTTCA |
| Mp4CL1-At-F1 | AGTTACGCAGGCTTCCTACG |
| Mp4CL1-At-R1 | AGAGAACCACCTTGACGGAG |
| AtActin-F2 | TATTGTGCTGGATTCTGGTG |
| AtActin-R2 | GTGCTGTGATTTCTTTGCTC |
| attB1-Mp4CL1 | GGGGACAAGTTTGTACAAAAAAGCAGGCTTAACCATGGCGCCTACAATGGCTCC |
| attB2-Mp4CL1-GFP | GGGGACCACTTTGTACAAGAAAGCTGGGTCAACGAGGTTTCTTAAGTCTT |
| attB2-Mp4CL1-OE | GGGGACCACTTTGTACAAGAAAGCTGGGTCCTAAACGAGGTTTCTTAAGT |
| attB1-Mp4CL2 | GGGGACAAGTTTGTACAAAAAAGCAGGCTTAACCATGGGTTACGAGAAGAGTGG |
| attB2-Mp4CL2-GFP | GGGGACCACTTTGTACAAGAAAGCTGGGTCCAATTTCGAAGTAGGAGCCA |
| Mp4CL1-BglII-F | GAAGATCTCATGGCGCCTACAATGGCTCC |
| Mp4CL1-KpnI-R | CGGGGTACCCTAAACGAGGTTTCTTAAGT |
| MpCHS-BamHI-F | CGCGGATCCGATGAGCAGGTCCCGACTCAT |
| MpCHS-HindIII-R | CCCAAGCTTTTACACTCTCTCAGCCTGCT |
| AtCHS-BamHI-F | CGCGGATCCGATGGTGATGGCTGGTGCTTC |
| AtCHS-NotI-R | ATAAGAATGCGGCCGCTTAGAGAGGAACGCTGTGCA |
| ScCHS1-HindIII-F | CCCAAGCTTATGGCCAGCGCCACCATCCC |
| ScCHS1-NotI-R | ATAAGAATGCGGCCGCTCATACCAGGGGCACGCTGC |

|  |  |
| --- | --- |
|  |  |
|  |  |
|  |  |
|  |  |
|  |  |
|  |  |
|  |  |
|  |  |
|  |  |
|  |  |
|  |  |
|  |  |
|  |  |
|  |  |
|  |  |

|  |  |  |  |
| --- | --- | --- | --- |
|  |  |  |  |
|  |  |  |  |
|  |  |  |  |
|  |  |  |  |
|  |  |  |  |
|  |  |  |  |
|  |  |  |  |
|  |  |  |  |
|  |  |  |  |
|  |  |  |  |
|  |  |  |  |
|  |  |  |  |
