# Supplementary material for: Molecular cloning and functional analysis of 4-coumarate: CoA ligases from Marchantia paleacea and their roles in lignin and flavanone biosynthesis
Source: PLoS One. 2024 Jan 8;19(1):e0296079. doi: 10.1371/journal.pone.0296079 (PMC10773943; doi:10.1371/journal.pone.0296079)
Supplement: S3 Table — (DOC) [file pone.0296079.s007.doc]

**Supporting information**

|  |  |
| --- | --- |
|  |  |
|  |  |
|  |  |
|  |  |
|  |  |
|  |  |
|  |  |
|  |  |
|  |  |
|  |  |
|  |  |
|  |  |
|  |  |
|  |  |
|  |  |
|  |  |
|  |  |
|  |  |
|  |  |
|  |  |
|  |  |
|  |  |

|  |  |
| --- | --- |
|  |  |
|  |  |
|  |  |
|  |  |
|  |  |
|  |  |
|  |  |
|  |  |
|  |  |
|  |  |
|  |  |
|  |  |
|  |  |
|  |  |
|  |  |
|  |  |
|  |  |
|  |  |
|  |  |
|  |  |
|  |  |
|  |  |
|  |  |
|  |  |
|  |  |
|  |  |
|  |  |
|  |  |
|  |  |
|  |  |
|  |  |
|  |  |
|  |  |
|  |  |
|  |  |
|  |  |

**S3 Table. Plasmids and strains were used in this study.**

| Plasmids or strains | Description |
| --- | --- |
| Plasmids |  |
| pET32a | T7 promoter, F1 ori, Amp^R^ |
| pCDFDuet-1 | Double T7 promoters, CDF ori, Sm^R^ |
| pCDFDuet-1-MpCHS | pCDFDuet-1 carrying MpCHS |
| pCDFDuet-1-AtCHS | pCDFDuet-1 carrying AtCHS |
| pCDFDuet-1-ScCHS1 | pCDFDuet-1 carrying ScCHS1 |
| p1 | pCDFDuet-1 carrying MpCHS and Mp4CL1 |
| p2 | pCDFDuet-1 carrying AtCHS and Mp4CL1 |
| p3 | pCDFDuet-1 carrying ScCHS1 and Mp4CL1 |
| *E. coli* Strains |  |
| *E. coli* DH5α |  |
| *E. coli* BL21 |  |
| E1 | BL21(DE3) carrying p1 |
| E2 | BL21(DE3) carrying p2 |
| E3 | BL21(DE3) carrying p3 |

|  |  |  |  |
| --- | --- | --- | --- |
|  |  |  |  |
|  |  |  |  |
|  |  |  |  |
|  |  |  |  |
|  |  |  |  |
|  |  |  |  |
|  |  |  |  |
|  |  |  |  |
|  |  |  |  |
|  |  |  |  |
|  |  |  |  |
|  |  |  |  |
