# Supplementary material for: Molecular cloning and functional analysis of 4-coumarate: CoA ligases from Marchantia paleacea and their roles in lignin and flavanone biosynthesis
Source: PLoS One. 2024 Jan 8;19(1):e0296079. doi: 10.1371/journal.pone.0296079 (PMC10773943; doi:10.1371/journal.pone.0296079)
Supplement: S4 Table — (DOC) [file pone.0296079.s008.doc]

**Supporting information**

|  |  |
| --- | --- |
|  |  |
|  |  |
|  |  |
|  |  |
|  |  |
|  |  |
|  |  |
|  |  |
|  |  |
|  |  |
|  |  |
|  |  |
|  |  |
|  |  |
|  |  |
|  |  |
|  |  |
|  |  |
|  |  |
|  |  |
|  |  |
|  |  |

|  |  |
| --- | --- |
|  |  |
|  |  |
|  |  |
|  |  |
|  |  |
|  |  |
|  |  |
|  |  |
|  |  |
|  |  |
|  |  |
|  |  |
|  |  |
|  |  |
|  |  |
|  |  |
|  |  |
|  |  |
|  |  |
|  |  |
|  |  |
|  |  |
|  |  |
|  |  |
|  |  |
|  |  |
|  |  |
|  |  |
|  |  |
|  |  |
|  |  |
|  |  |
|  |  |
|  |  |
|  |  |
|  |  |

|  |  |
| --- | --- |
|  |  |
|  |  |
|  |  |
|  |  |
|  |  |
|  |  |
|  |  |
|  |  |
|  |  |
|  |  |
|  |  |
|  |  |
|  |  |
|  |  |
|  |  |

**S4 Table. The conversion rate generated by the four hydroxycinnamic acids to produce naringenin, pinocembrin, eriodictyol, and homoeriodictyol at the 300 μM substrate concentration in the TB medium.**

| Strain | Substrate | Metabolites | Conversion rate (%) |
| --- | --- | --- | --- |
| E1 | *p*-Coumaric acid | Naringenin | 60.8 |
|  | Cinnamic acid | Pinocembrin | 5.6 |
|  | Caffeic acid | Eriodictyol | 0 |
|  | Ferulic acid | Homoeriodictyol | 2.6 |
| E2 | *p*-Coumaric acid | Naringenin | 23.1 |
|  | Cinnamic acid | Pinocembrin | 4.6 |
|  | Caffeic acid | Eriodictyol | 0 |
|  | Ferulic acid | Homoeriodictyol | 0 |
| E3 | *p*-Coumaric acid | Naringenin | 0 |
|  | Cinnamic acid | Pinocembrin | 0 |
|  | Caffeic acid | Eriodictyol | 0 |
|  | Ferulic acid | Homoeriodictyol | 0 |
